# Synthesis, Structure, and Electrophysical and Electrochemical Properties of Novel Composite La_0.9_MnO_3_-LaFeO_3_

**DOI:** 10.3390/molecules30010132

**Published:** 2024-12-31

**Authors:** Mukhametkali Mataev, Zamira Sarsenbaeva, Bahadir Keskin, Marzhan Nurbekova, Amangeldi Meldeshov, Zhanar Tursyn, Karima Seitbekova

**Affiliations:** 1 Department of Chemistry, Faculty of Natural Sciences, Kazakh National Women’s Teacher Training University, Gogol 114/1, Almaty 050000, Kazakhstan; mataev.m@qyzpu.edu.kz (M.M.);; 2 Department of Chemistry, Faculty of Arts & Science, Yildiz Technical University, Istanbul TR34220, Turkey; bahadirkeskin@gmail.com

**Keywords:** double perovskite, synthesis, Pechini method, electroconductivity, HER

## Abstract

This article presents the synthesis, electrophysical, and catalytic properties of a La_0.9_MnO_3_–LaFeO_3_ nanocomposite material. The nanocomposite was synthesized via the sol–gel (Pechini) method. X-ray diffraction (XRD) analysis revealed a polycrystalline, biphasic perovskite structure combining both hexagonal and cubic symmetry. The microstructure and elemental composition, examined using field emission scanning electron microscopy (FESEM), indicated an average particle size of approximately 186.9 nm. The composite exhibits semiconducting behavior within the temperature ranges of 293–323 K and 343–393 K. Developing electrocatalysts free of precious metals for the hydrogen evolution reaction (HER) is increasingly important to facilitate the production of hydrogen from renewable sources. In this study, the conductive La_0.9_MnO_3_–LaFeO_3_ composite was deposited on graphite and, for the first time, evaluated as an electrocatalyst for HER in acidic media. The resulting composite films were tested using linear sweep voltammetry (LSV) and electrochemical impedance spectroscopy (EIS) in a glassy carbon electrode (GCE) setup, providing insights into their potential as effective, cost-efficient electrocatalysts.

## 1. Introduction

In view of the depletion of natural resources and escalating environmental pollution, the development, conversion, and storage of alternative energy sources have gained significant attention [1]. Recently, ABO_3_-type perovskites (where A is a rare-earth or alkaline-earth metal and B is a transition metal) have emerged as promising candidates for energy conversion and storage in metal–air batteries and solid oxide fuel cells, owing to their high catalytic activity [2,3,4,5]. Additionally, these materials exhibit structural and compositional flexibility, enabling various modifications [6,7]. The presence of ferroelectric (FE) and magnetic properties in multiferroic perovskites further broadens their range of potential applications [8,9,10,11]. Due to these versatile characteristics, perovskite oxides are utilized in heterogeneous catalysis [12,13], sensing [14], supercapacitors [15,16], fuel cells [17], and metal–air batteries [18,19].

Electrolysis of water is an environmentally friendly method for sustainable hydrogen production that both prevents pollution and allows for the recycling of resources [20]. Although Pt-based materials are effective catalysts for the oxygen evolution reaction (OER) and hydrogen evolution reaction (HER), their limited durability, modest efficiency, and high cost impede large-scale implementation. Consequently, developing cost-effective electrocatalysts with high catalytic performance is of critical importance. In recent years, transition metal oxides in the form of double perovskites (ABO_3_–AB′O_3_) have attracted considerable attention as electrocatalysts due to their diverse oxygen valence states, favorable redox properties, high electrochemical activity, and cost-effectiveness [21].

Among ABO_3_-type perovskites, LaMnO_3_ is well known for its colossal magnetoresistance [22,23]. It has also garnered interest as a material for energy conversion and storage [6], notably as a noble metal-free oxide catalyst [24] and as a promising electrode material for supercapacitors [25]. However, the inherently poor electron transport in LaMnO_3_ limits its practical application. Interestingly, it has been reported [5] that the conductivity (σ) of La_0.9_MnO_3_ at room temperature is nearly an order of magnitude greater than that of LaMnO_3_, making it an appealing candidate for enhancing multifunctional properties [26].

Complex oxides of the type AFeO_3_ (A = rare-earth metal), known as orthoferrites, are also of considerable interest [27]. Among these, LaFeO_3_-based compounds are widely recognized for their applications as sensors [28], electrode materials in solid oxide fuel cells [29], and photocatalysts [30,31]. These p-type semiconductors possess unique physical and chemical properties [32]. LaFeO_3_ crystallizes in an orthorhombically distorted perovskite structure (space group Pnma) and exhibits canted antiferromagnetism, arising from superexchange interactions between neighboring Fe^3+^ ions [27].

This study focuses on the preparation of a highly concentrated dual-phase nanomaterial composed of lanthanum manganite-ferrite within a single system. A La_0.9_MnO_3_–LaFeO_3_ nanocomposite was synthesized via the sol–gel (Pechini) method, achieving optimal phase distribution and a high degree of crystallinity. The presence of transition metals in this unique combination of phases fosters a synergistic effect that promotes both electronic and ionic conductivity (Mn^3+^/Mn^4+^ and Fe^3+^/Fe^4+^). The electrocatalytic activity of this dual-phase perovskite nanocomposite was investigated for the HER, a reaction of growing significance as a sustainable energy source. The aim of this work is to synthesize a novel dual-phase lanthanum manganite-ferrite material and to evaluate its electrical conductivity and activity in the HER.

## 2. Results and Discussion

### 2.1. Preparation of La_0.9_MnO_3_-LaFeO_3_ Nanocomposite

**Figure 1:**
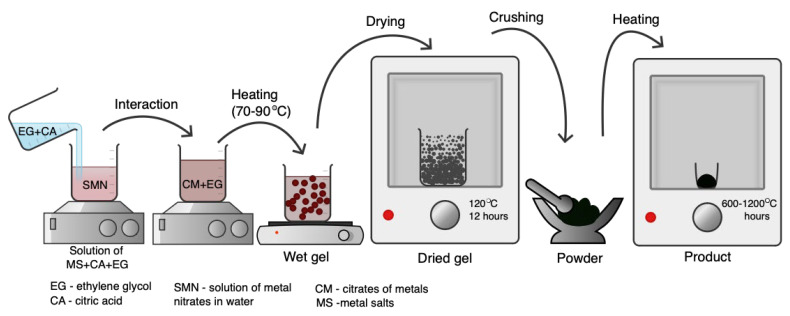
Steps of preparation of La_0_._9_MnO_3_-LaFeO_3_ particles by Pechini-type sol–gel method.

Manganites can be synthesized using different sol–gel methods; in this study, the Pechini method was chosen to produce a double-phase composite [33] (Figure 1). During the synthesis of La_0.9_MnO_3_-LaFeO_3_, varying precursor ratios were evaluated, including 9:1, 87:13, and 85:15. The 87:13 composition was selected for detailed investigation due to its favorable yield and reproducibility. For the synthesis, stoichiometric quantities of the precursors were measured as follows: 3.77 g of La(NO_3_)_3_·6H_2_O, 3.51 g of Fe(NO_3_)_3_·9H_2_O, and 1.58 g of Mn(NO_3_)_2_·xH_2_O. To prepare salt solutions, 10 mL of distilled water was added. To facilitate the synthesis, 2.0 g of citric acid (added in a 1:1.5 molar ratio to the total metal cations) and 2.72 mL of ethylene glycol (density: 1.1 g/mL) were included. Citric acid acted as a chelating agent, while ethylene glycol promoted polymerization, ensuring the formation of a homogeneous gel and contributing to the successful synthesis of the desired phase. The resulting gel was dried at 120 °C for 12 h and the porous product was milled and fired at 600–1200 °C for 6 h. As a result, a powder of perovskite-like biphasic nanocomposite was synthesized (Figure S1). The obtained product was analyzed by FESEM and XRD methods for the elemental composition and crystal structure of the complex oxide compound. The electrical conductive properties of the nanomaterial were determined.

### 2.2. X-Ray Diffraction (XRD) Analysis

For the X-ray diffraction (XRD) analysis, we used a Rigaku MiniFlex 600 X-ray diffraction system from Rigaku Corporation in Tokyo, Japan. The system utilized CuKα radiation with a range of 2θ 3–120 °C, a step size of 2θ 0.01–0.02°, and a time per step of 0.3–0.5 °C. A nickel monochromator was employed to capture the diffraction, and the data were analyzed using the PDXL2 databases. The average crystal sizes of La_0.9_MnO_3_-LaFeO_3_ were calculated using Debye–Scherrer’s formula.

**Figure 2:**
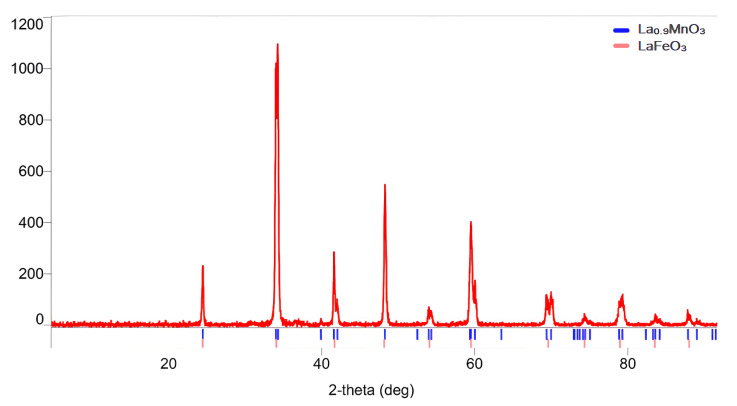
XRD of the La_0.9_MnO_3_-LaFeO_3_.

The diffraction peaks in Figure 2 demonstrate that double-phase manganite and ferrite of lanthanum with sample purity and phase percentage of 87:13 were produced.

Table 1 presents the syngonic types, lattice parameters (a, b, c), space group, X-ray density, and calculated average crystal sizes (D) determined based on X-ray diffraction (XRD) data. It is determined whether the diffraction peaks (PDXL 2) of the synthesized sample are indexed in the perovskite-like structure. Only sharp diffraction peaks show high crystallinity in all samples. The average crystallite size, D, was calculated by Scherrer’s equation [34].

D = k λ/β cos θ(1)

In this equation, the value of the shape factor (k) is 0.94 and λ = 1.5406 Å, β is the line broadening at half of the maximum intensity (FWHM), and θ is the Bragg angle (in radians). The crystal sizes at high peak values correspond to 55.29 nm for lanthanum manganite and 54.96 nm for lanthanum ferrite.

### 2.3. FTIR Spectroscopy Analysis

Fourier-transform infrared (FT-IR) spectroscopy was conducted using a Bruker ALPHA instrument (Ettlingen, Germany) under ambient conditions. The analysis was performed within the spectral range of 4000–400 cm^−1^, employing KBr pellets as the medium. The resolution was set to 1 cm^−1^, and to enhance the signal-to-noise ratio, 32 scans were averaged for each measurement.

The surface chemistry of nanostructured materials significantly influences their physicochemical properties, primarily due to the presence of surface defects and structural heterogeneity. In this study, FT-IR spectroscopy was employed to investigate the chemical bonding between Mn–O and Fe–O atoms in nanostructured La_0.9_MnO_3_-LaFeO_3_, providing valuable insights into its structural and compositional attributes.

**Figure 3:**
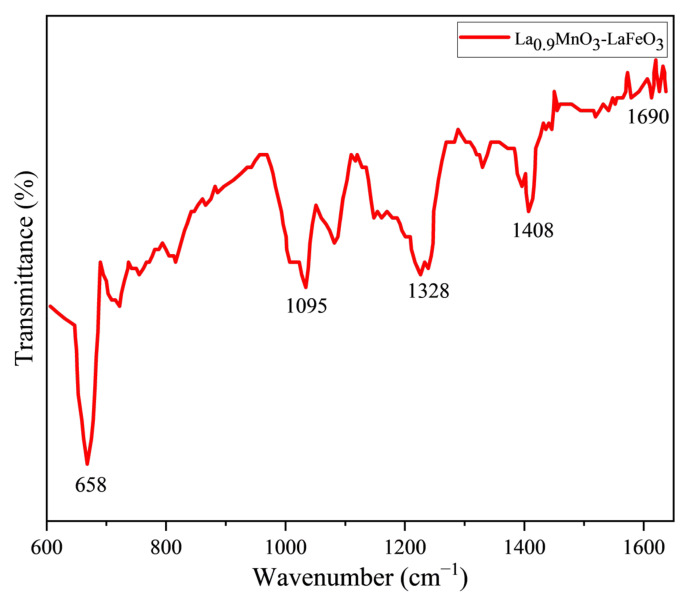
FT-IR spectra for La_0.9_MnO_3_-LaFeO_3_.

Figure 3 shows the FT-IR spectrum of La_0.9_MnO_3_-LaFeO_3_ nanoparticles, with significant absorption bands at 658, 1095, 1328, 1408, and 1690 cm^−1^. The band at 658 cm^−1^ corresponds to the stretching mode of Mn-O-Mn and Fe-O-Fe bonds, associated with the MnO_6_ and FeO_6_ octahedra in ABO_3_-type perovskites [35]. The band near 1690 cm^−1^ is attributed to O-H (La(OH)) bonds, which may be linked to the hygroscopic nature of the compounds. The band at 1328 cm^−1^ likely corresponds to specific vibrations of the oxide structures. The bands at 1408 cm^−1^ and 1095 cm^−1^ are associated with carboxylate (COO-) and C-O (carbon) bonds, respectively, which could be related to residues from the sol–gel synthesis process [35,36].

### 2.4. FESEM Analysis

The finely powdered La_0.9_MnO_3_-LaFeO_3_ was subjected to FESEM to examine its surface morphology and to determine its elemental composition. The analysis was performed using a Thermo Scientific Apreo 2 S LoVac (Waltham, MA, USA). The elemental composition was assessed through energy-dispersive X-ray spectroscopy (EDS), which was integrated with the SEM instrument. FESEM provided high-resolution surface measurements at 500 nm. This method enabled detailed visualization of the morphology and facilitated the identification of the elemental distribution within the synthesized phases, offering critical insights into the structural and compositional attributes of the material.

**Figure 4:**
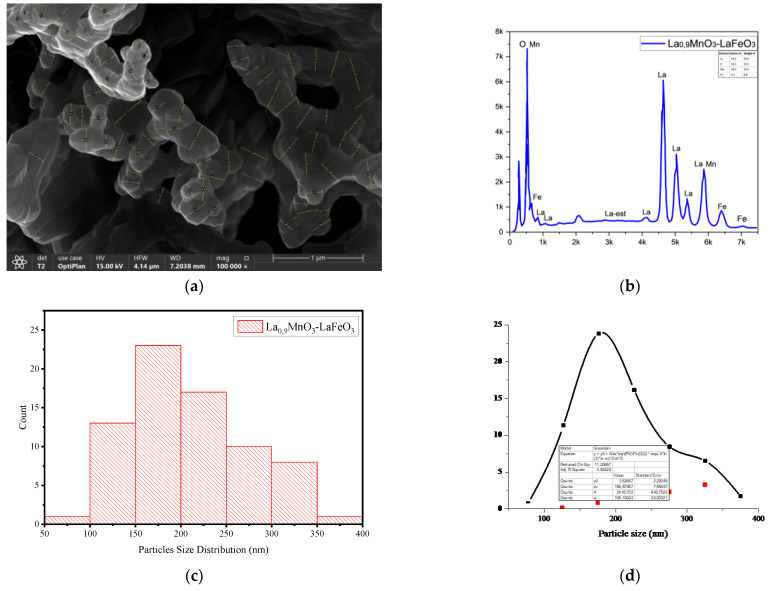
Particle size distribution histograms and FESEM micrographs of manganite-ferrite sample. (**a**) FESEM micrograph; (**b**) EDS spectrum; (**c**) Particle size distribution; (**d**) Particle size.

Figure 4 (Figures S2 and S3) depicts FESEM-EDAX images (a,b) and particles size distribution (c,d) of La_0.9_MnO_3_-LaFeO_3_ sample. The values of polycrystal surface size were measured using ImageJ program (https://imagej.net/ij/). It was determined that the element distribution and the average crystal size indicate values of 186.87 nm.

### 2.5. Preparation of a Tablet for Electrophysical Measurement

Electrophysical properties were measured according to the methods [37,38]. The research on electrophysical properties (dielectric constant and electrical resistivity) was carried out by measuring the electrical capacitance of the samples on a commercially available LCR-800 instrument (Taiwan) at an operating frequency of 1 kHz in continuous thermostat mode in dry air with each fixed temperature maintenance time. The research on electrophysical properties (dielectric permittivity and electrical resistance) was carried out by measuring the electrical capacity of the samples on a commercially available device LCR-800 (Taiwan) at an operating frequency of 1 kHz continuously in dry air in thermostatic mode with holding time at each fixed temperature. Flat-parallel specimens in the form of disks with a diameter of 10 mm and thickness of 2–6 mm with binder additive (~1.5%) were prefabricated. Pressing was carried out under pressure of 20 kg/cm^2^. The obtained disks were fired in the laboratory furnace at 400 °C for 6 h. Then thorough double-sided grinding was carried out.

Dielectric permittivity was determined from the electrical capacity of the sample at known values of sample thickness and electrode surface area. A Sawyer–Tower scheme was used to obtain the relationship between the electrical induction D and the electric field strength E. Visual observation of D (E of the hysteresis loop) was performed on a C1-83 oscilloscope with a voltage divider consisting of a 6 mOhm and 700 kOhm resistance and a 0.15 µF reference capacitor. The frequency of the oscillator was 300 Hz. In all temperature studies, the samples were placed in an oven and the temperature was measured with a chromel-alumel thermocouple connected to a B2-34 voltmeter with an error of ±0.1 mV. The rate of temperature change was ~5 K/min. The value of dielectric permittivity at each temperature was determined by the following formula:

(2) $$\varepsilon =\frac{C}{C_{0}}$$

where $C_{0}=\frac{\varepsilon_{0}\cdot S}{d}$—is the capacitance of the capacitor without the investigated substance (air).

The calculation of the forbidden band width (ΔE) of the investigated substance was determined by the formula [39]:

(3) $$\Delta E=\frac{2kT_{1}T_{2}}{0.43\left(T_{2}-T_{1}\right)}\mathrm{lg}\frac{R_{1}}{R_{2}},$$

where K is the Boltzmann constant equal to 8.6173303·10^−5^ eV·K^−1^ t, R_1_ is the resistance at T_1_, and R_2_ is the resistance at T_2_.

**Figure 5:**
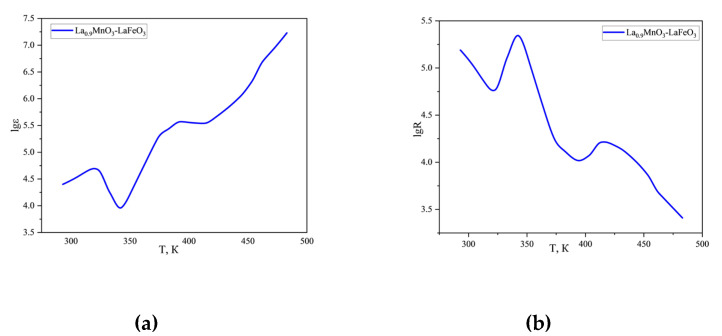
Dependence of dielectric permittivity (**a**) and electrical resistivity (**b**) of La_0.9_MnO_3_-LaFeO_3_ on temperature and frequency equal to 1 kHz.

Electrophysical measurements of La_0.9_MnO_3_-LaFeO_3_ in the range 293–483 K and frequencies equal to 1, 5, and 10 kHz were carried out on the LCR-800 setup (Table 2, Figure 5).

The data of Table 2 and Figure 5 show that the value of ɛ equal to 25,182 at 293 K (1 kHz) reaches gigantic values up to 1.72 × 10^7^ when the temperature is increased to 483 K. When the frequency is increased to 10 kHz, the value of ɛ decreases, remaining relatively high at 483 K, equal to 5.35·10^5^.

The study of temperature dependence of electrical resistivity on temperature of material 2 shows a complex character of conductivity: in the interval 293–323 K—semiconducting, at 323–343 K—variable conductivity, at 343–393 K—semiconducting, 393–413 K—metallic and at 413–483 K—again semiconducting (Table 3).

The forbidden band width of La_0.9_MnO_3_-LaFeO_3_ in the interval 293–323 K, 343–393 K, and 413–483 K are equal to 1.38, 1.44, and 1.41 eV, respectively, and this can be attributed to narrow bandgap semiconductors.

### 2.6. Electrochemical Measurement

#### 2.6.1. Preparation of Working Electrode

The prepared electrocatalysts were tested in a conventional triple electrode (reference electrode: Ag/AgCl; counter electrode: platinum plate; working electrode: glassy carbon) system setup in an acidic solution of 0.5 M sulfuric acid using a Gamry electrochemical workstation (Reference 600 Potentiostat) to evaluate their electrocatalytic activity for bifunctional electrocatalytic activity. A glassy carbon electrode (GCE: 0.0314 cm^−2^) was used as the working electrode. To clean the working electrode, the GCE was polished with alumina powder (0.05 μm) and then sonicated in a mixture of EtOH and H_2_O (1:3) for 5 min. To prepare homogenized ink, 5 mg of La_0.9_MnO_3_-LaFeO_3_ and 2 mg of carbon black composite was dispersed in 1 mL of H_2_O and further sonicated for 30 min. Then 15 μL of homogenized catalytic ink and 10 μL of Nafion-117 were dripped onto the cleaned GCE surface and allowed to dry at room temperature, respectively (Figure S4). Electrochemical impedance spectroscopy (EIS) and LSV were recorded for HER in 0.5 mol/L H_2_SO_4_ in the corresponding potential range [40].

#### 2.6.2. Linear Sweep Voltammetry Analysis

**Figure 6:**
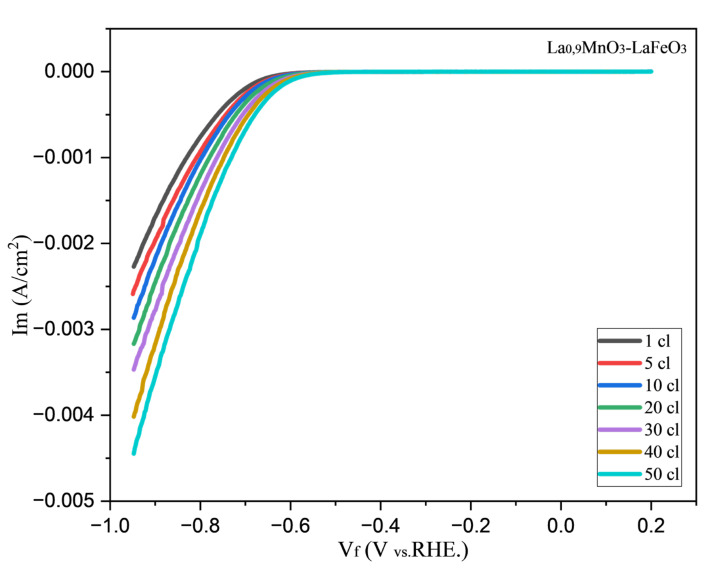
LSV curves for the nanocomposite La_0.9_MnO_3_-LaFeO_3_.

LSV curves for HER with La_0.9_MnO_3_-LaFeO_3_ nanocomposites are shown in Figure 6. The polarization method is a systematic and effective tool to investigate the electrochemical activity of electrocatalysts. These curves were recorded from 0.2–0.95 V vs. Ag/AgCl (reference electrode) at a scan rate of 9.99 mV/s in 0.5 M H_2_SO_4_ using a three-probe electrode system.

Linear sweep voltammetric curves were obtained to determine the activity of La_0.9_MnO_3_-LaFeO_3_ nanocomposite. A total of 50 cycles of voltammetric curves were recorded, with the curves from cycles 1, 5, 10, 20, 30, 40, and 50 selected for comparison. As a result of studying the curves, it can be seen that the number of loops with each rope increased the current value in Figure 5. In the first cycle, the current value was increased to 0.0022 A and in cycle 50 to 0.0045 A. From all the curves, we can observe that the catalyst affects the hydrogen release. That is, the results showed that the activity of La_0.9_MnO_3_-LaFeO_3_ nanocomposite catalyst increased with increasing number of cycles.

The HER activity of this electrocatalyst can be based on several factors, such as, for the deposited electrocatalyst in solution, charge carrier resistance, specific surface area of new active sites, microstrains in the electrocatalyst, surface defects, and oxygen gaps.

To further evaluate the electrocatalytic properties of the HER materials, the charge transfer resistance (Rct) was measured using potentiostatic electrochemical impedance spectroscopy (EIS), in which an alternating voltage was applied to the electrode and the resulting current was measured.

The method of electrochemical impedance spectroscopy was used to study the surface layer of the electrode. An electrochemically equivalent cell is used to fully characterize the processes occurring in the electrode and electrolyte region.

#### 2.6.3. Electrochemical Impedance Spectroscopy

EIS is an effective method to determine the electrochemical characteristics of surface catalysts, packing interfaces, etc. Electrocatalytic water splitting is one of the industries specializing in the production of high purity hydrogen where EIS is used to correlate trends that measure charge transfer activity (Rct). An electrochemically equivalent cell consists of the following elements: solution (Rs), charge transfer resistance (Rct) and Warburg (W) elements, double electrical layer capacitance (Cdl), and constant phase (CPE Electrochemical impedance spectroscopy (EIS) was performed for La_0.9_MnO_3_-LaFeO_3_ at various overpotentials in 0.5 M H_2_SO_4_. The data were analyzed using the simplified equivalent circuit depicted in the inset, with the fitting results represented by solid lines.

**Figure 7:**
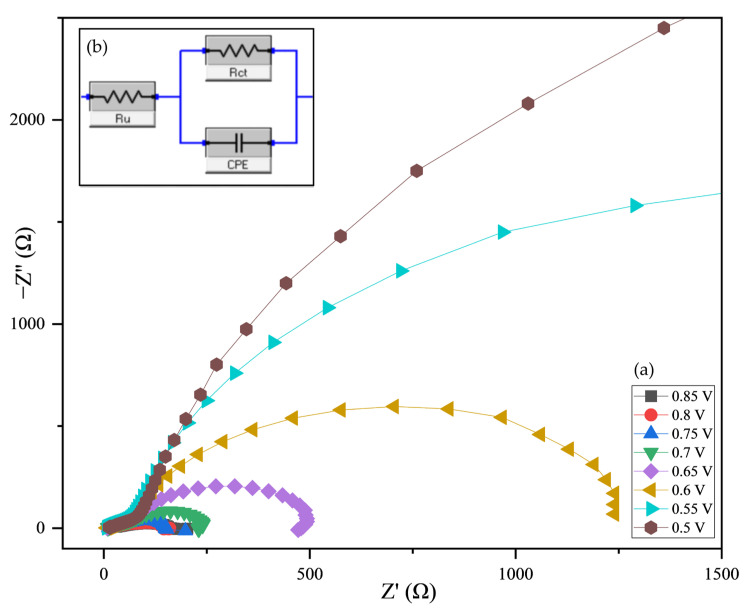
Nyquist plot (**a**) and electrical equivalent circuits (Rct) (**b**) for La_0.9_MnO_3_-LaFeO_3_.

Figure 7 shows the Nyquist plot of the impedance of La_0.9_MnO_3_-LaFeO_3_ at different applied voltages, with the voltage increasing from 0.5 V to 0.85 V. The graph shows that the impedance of the material decreases as the applied voltage increases. The graphs with higher voltages indicate that the impedance of the material is increasingly dominated by the resistive component at higher frequencies. This is attributed to the fact that the material undergoes a phase transition at high voltages. The method of electrochemical impedance spectroscopy was used to study the surface layer of the electrode. An electrochemically equivalent cell is used to fully characterize the processes occurring in the electrode and electrolyte region.

## 3. Materials and Methods

The following reagents were used: manganese (II) nitrate (Mn(NO_3_)_2_∙xH_2_O, Buchs, Switzerland); iron (III) nitric acid crystalline hydrate with 9-water salt (Fe(NO_3_)_3_∙9H_2_O, TU 6-09-02-553-96); lanthanum (III) nitrate with water 6-crystalline hydrate (La(NO_3_)_3_∙6H_2_O, TU 6-09-4676-83); citric acid (C_6_H_8_O_7_) (GOST 908-79) and ethylene glucol (C_2_H_6_O_2_) (GOST 10164-75).

The instruments and methods of measurement used were as follows: laboratory agate, (diameter—13 cm (130 mm) (QIANGFU, ASIN, B0DNB1MQ28, Origin, China), and laboratory furnace “SNOL” (UAB Umey, Utena, Lithuania). X-ray phase method (Miniflex 600 Rigaku, Tokyo, Japan) and field emission scanning electron microscope (Thermo Scientifıc Apreo 2 S LoVac, Waltham, MA, USA) were used to determine the phase composition, and a commercially available LCR-800 (Taipei Hsien, Taiwan) was used to determine the electrophysical properties. IR spectra were recorded on a Bruker ALPHA FTIR spectrometer in the range of 400–4000 cm^−1^. Potentiostat reference 600 was used to obtain LSV and EIS data.

## 4. Conclusions

The La_0.9_MnO_3_–LaFeO_3_ nanopowder was synthesized via the Pechini (sol–gel) method. The crystal structure, elemental composition, and morphology of the double-phase perovskite were examined using XRD, FTIR, and FESEM. These analyses confirmed that the resulting biphasic nanocomposite exhibits both hexagonal and cubic symmetries. Crystallographic evaluation revealed that the lanthanum manganite phase adopts a hexagonal unit cell (Z = 6) with lattice parameters a = b = 5.52 Å and c = 13.37 Å, while the LaFeO_3_ phase crystallizes into a cubic perovskite-like structure (Z = 1) with a = b = c = 3.89 Å. The nanoscale size and elemental composition of the product further validated its successful formation.

Electrophysical measurements indicated that the nanomaterial displays semiconducting behavior across three distinct temperature ranges: 293–323 K, 343–393 K, and 413–483 K. The study also systematically investigated how the thickness of the La_0.9_MnO_3_–LaFeO_3_ composite film and the electrodeposition process on a GCE influence the electrochemical properties, as analyzed by EIS. The resulting La_0.9_MnO_3_–LaFeO_3_/GCE electrode, noted for its stability and cost-effectiveness, demonstrates potential as an efficient electrocatalyst for hydrogen production from water.

To generate LSV curves, measurements were recorded after 1, 5, 10, 20, 30, 40, and 50 cycles. The LSV results show that the electrocatalytic activity of the double-phase nanocomposite catalyst increases with the number of cycles. However, at potential values of 0.85–0.6 V, EIS data indicate that electrolyte resistance and double-layer capacitance dominate, rather than charge transfer resistance, suggesting comparatively low activity of the nanocomposite under these conditions.

## Figures and Tables

**Table 1:** Results of quantitative analysis of the crystal lattice.

N	Name of Compound	Types of Syngony	a, Å	b, Å	c, Å	Vc., (Å^3^)	Z	D (Crystal Size, nm)	Space Group	Density (ρx-Ray, g/cm^3^)
1	La_0.9_MnO_3_	hexagonal	5.52	5.52	13.37	354.10	6	55.29	R-3c (161)	6.40
2	LaFeO_3_	cubic	3.89	3.89	3.89	58.99	1	54.96	Pm-3m (211)	6.76

**Table 2:** Electrophysical measurements.

T, K	C, nF	R, Om	ε	lgε	lgR
1	2	3	4	5	6
Measurement frequency 1 kHz					
293	8.74	155,100	25,152	4.40	5.19
303	11.34	110,800	32,649	4.51	5.04
313	15.31	72,760	44,092	4.64	4.86
323	16.38	59,390	47,147	4.67	4.77
333	6.07	127,900	17,480	4.24	5.11
343	3.26	220,500	9397	3.97	5.34
353	7.73	103,600	22,268	4.35	5.02
363	21.69	41,820	62,430	4.80	4.62
373	60.36	18,220	173,769	5.24	4.26
383	95.42	12,880	274,707	5.44	4.11
393	127.93	10,480	368,290	5.57	4.02
403	123.17	11,630	354,586	5.55	4.07
413	121.78	16,310	350,585	5.54	4.21
423	159.34	15,620	458,714	5.66	4.19
433	237.49	13,060	683,695	5.83	4.12
443	373.64	10,250	1,075,649	6.03	4.01
453	705.10	7483	2,029,868	6.31	3.87
463	1789.50	4696	5,151,679	6.71	3.67
473	3192.40	3486	9,190,399	6.96	3.54
483	5962.60	2582	17,165,353	7.23	3,41
Measurement frequency 5 kHz					
293	0.90	122,800	2600	3.42	5.09
303	1.42	87,570	4080	3.61	4.94
313	2.28	56,080	6565	3.82	4.75
323	2.32	51,730	6669	3.82	4.71
333	0.86	110,100	2480	3.39	5.04
343	0.52	155,000	1502	3.18	5.19
353	1.36	76,840	3903	3.59	4.89
363	4.27	29,990	12,306	4.09	4.48
373	10.71	15,030	30,835	4.49	4.18
383	16.87	10,830	48,566	4.69	4.03
393	22.28	9431	64,140	4.81	3.97
403	21.27	10,300	61,241	4.79	4.01
413	19.59	15,120	56,419	4.75	4.18
423	24.67	14,240	71,044	4.85	4.15
433	33.16	12,400	95,454	4.98	4.09
443	49.41	9486	142,252	5.15	3.98
453	84.09	6989	242,084	5.38	3.84
463	190.12	4512	547,324	5.74	3.65
473	319.55	3359	919,932	5.96	3.53
483	553.74	2487	1,594,127	6.20	3.40
Measurement frequency 10 kHz					
293	0.41	98,840	1166	3.07	4.99
303	0.64	71,080	1854	3.27	4.85
313	1.11	45,830	3200	3.51	4.66
323	0.93	51,020	2663	3.43	4.71
333	0.35	102,900	999	3.00	5.01
343	0.27	119,400	764	2.88	5.08
353	0.69	60,850	2000	3.30	4.78
363	2.34	23,840	6734	3.83	4.38
373	5.17	13,150	14,880	4.17	4.12
383	8.16	9711	23,481	4.37	3.99
393	10.04	8959	28,915	4.46	3.95
403	9.93	9640	28,587	4.46	3.98
413	8.58	14,620	24,692	4.39	4.16
423	10.52	13,610	30,277	4.48	4.13
433	13.79	11,590	39,696	4.60	4.06
443	19.89	8957	57,277	4.76	3.95
453	33.25	6646	95,707	4.98	3.82
463	67.49	4299	194,293	5.29	3.63
473	113.55	3229	326,892	5.51	3.51
483	185.71	2430	534,629	5.73	3.39

**Table 3:** Values of the forbidden band at different temperatures.

(a) Calculation of the forbidden band width in the interval 293–323 K:	
T, K	lg R
293	5.19
323	4.77
$\Delta E\;=\frac{2\;\times \;0.000086173\;\times \;293\;\times \;323}{0.43\;\times \;(323\;\times 293)}\mathrm{lg}\frac{5.19}{4.77}=1.38\;\mathrm{eV}$	
(b) Calculation of the forbidden band width in the interval 343–393 K:	
T, K	lg R
343	5.34
393	4.02
$\Delta E=\frac{2\;\times \;0.000086173\;\times \;343\;\times \;393}{0.43\;\times \;(393\;\times \;343)}\mathrm{lg}\frac{5.34}{4.02}=1.44\;\mathrm{eV}$	
(c) Calculation of the forbidden band width in the interval 413–483 K:	
T, K	lg R
413	4.21
483	3.41
$\Delta E=\frac{2\;\times \;0.000086173\times 413\;\times \;483}{\;0.43\times \;(483\;\times \;413)}\mathrm{lg}\frac{4.21}{3.41}=1.41\;\mathrm{eV}$	

## Data Availability

The datasets used and/or analyzed during the present study are available from the corresponding author on reasonable request.

## Supplementary Materials

The following supporting information can be downloaded at: https://www.mdpi.com/article/10.3390/molecules30010132/s1, Figure S1: Photo images of the La_0.9_MnO_3_-LaFeO_3_ nanocomposite powder; Figure S2: EDS point analysis results (a) and XRSEM images (b) of La_0.9_MnO_3_-LaFeO_3_; Figure S3: Mapping analysis results of La_0.9_MnO_3_-LaFeO_3_ (a). Histogram of the particle size distribution of the samples determined from FESEM micrographs of manganite-ferrite composite (b,c); Figure S4: Photo images of the GCE modified with La_0.9_MnO_3_-LaFeO_3_ composite material.

## References

[1] Dunn B., Kamath H., Tarascon J.-M. (2011). Electrical Energy Storage for the Grid: A Battery of Choices. Science.

[2] Spaldin N.A., Ramesh R. (2019). Advances in magnetoelectric multiferroics. Nat. Mater..

[3] Shah N.A., Solanki P.S., Ravalia A., Kuberkar D.G. (2015). Size effects in magnetotransport in sol–gel grown nanostructured manganites. Appl. Nanosci..

[4] Henchiri C., Mnasri T., Benali A., Hamdi R., Dhahri E., Valente M.A., Costa B.F.O. (2020). Structural study and large magnetocaloric entropy change at room temperature of La_1−x_□_x_ MnO_3_ compounds. RSC Adv..

[5] Xie C., Shi L., Zhao J., Zhou S., Li Y., Guo J. (2017). Insight into the enhancement of transport property for oriented La_0.9_MnO_3_ films. J. Phys. Appl. Phys..

[6] Suntivich J., Gasteiger H.A., Yabuuchi N., Nakanishi H., Goodenough J.B., Shao-Horn Y. (2011). Design principles for oxygen-reduction activity on perovskite oxide catalysts for fuel cells and metal–air batteries. Nat. Chem..

[7] Suntivich J., May K.J., Gasteiger H.A., Goodenough J.B., Shao-Horn Y. (2011). A Perovskite Oxide Optimized for Oxygen Evolution Catalysis from Molecular Orbital Principles. Science.

[8] Suresh S., Vindhya P.S., Kavitha V.T. (2024). A comprehensive study of dielectric, magnetic and anticancerous properties of lanthanum manganite perovskite nanoparticles. J. Alloys Compd..

[9] Oumezzine M., Rostas A.M., Bocirnea A.E., Hlil E.K., Galca A.C. (2024). A-site K-doped lanthanum manganite nanocrystalline La_0.67_Ba_0.33_MnO_3_ for room-temperature micro-scale magnetic cooling. J. Alloys Compd..

[10] Tay F., Chaudhary S., He J., Marquez Peraca N., Baydin A., Fiete G.A., Zhou J., Kono J. (2023). Observation of colossal terahertz magnetoresistance and magnetocapacitance in a perovskite manganite. Optica.

[11] Patrin G.S., Mataev M.M., Abdraimova M.R., Tursinova Z.I., Kezdikbaeva A.T., Shiyan Y.G., Plekhanov V.G. (2021). Magnetic Properties of the DyMn_2_O_5_–Mn_3_O_4_ Nanoparticle Composite. Tech. Phys..

[12] Zhu J., Li H., Zhong L., Xiao P., Xu X., Yang X., Zhao Z., Li J. (2014). Perovskite oxides: Preparation, characterizations, and applications in heterogeneous catalysis. ACS Catal..

[13] Srivastava P., Kapoor I.P.S., Singh G. (2009). Nanoferrites: Preparation, characterization and catalytic activity. J. Alloys Compd..

[14] Ye L., Wu F., Xu R., Zhang D., Lu J., Wang C., Dong A., Xu S., Xue L., Fan Z. (2023). Face mask integrated with flexible and wearable manganite oxide respiration sensor. Nano Energy.

[15] Amalthi P., Vijaya J.J., Kumar R.T., Kennedy L.J., Bououdina M., Saravanakumar B. (2023). Microwave-aided fabrication of calcium-substituted DyMnO_3_ nanocomposites as prospective battery-type electrode material for supercapacitors. Mater. Sci. Eng. B.

[16] Vazhayil A., Vinaykumar R., Thomas J., Jeffery A.A., Hasan I., Thomas N., Yadav A.K., Ahn Y.H. (2024). The effect of B—Site cation on the supercapacitive properties of LaBO_3_ (B = Cr, Mn, Fe and Co) porous perovskites. J. Energy Storage.

[17] Lucia U. (2014). Overview on fuel cells. Renew. Sustain. Energy Rev..

[18] Qiu W., Lin Z., Xiao H., Zhang G., Gao H., Feng H., Lu X. (2021). Construction of chemical self-charging zinc ion batteries based on defect coupled nitrogen modulation of zinc manganite vertical graphene arrays. Mater. Adv..

[19] Yang F., Xie J., Rao D., Liu X., Jiang J., Lu X. (2021). Octahedral distortion enhances exceptional oxygen catalytic activity of calcium manganite for advanced Zn-Air batteries. Nano Energy.

[20] Sekar S., Sadhasivam S., Shanmugam A., Saravanan S., Pugazhendi I., Lee Y., Kim D.Y., Manikandan R., Chang S.C., Lee S. (2024). Enhanced bifunctional water electrolysis performance of spherical ZnMn_2_O_4_ nanoparticles. Int. J. Hydrogen Energy.

[21] Vishwakarma A.K., Hussain M., Verma S.K., Shukla V., Shaz M.A., Srivastava O.N. (2021). Synthesis and characterizations of graphene/Sm doped BiFeO_3_ composites photoanode for efficient photo-electrochemical water splitting. Int. J. Hydrogen Energy.

[22] Dagotto E. (2005). Complexity in Strongly Correlated Electronic Systems. Science.

[23] Jin S., Tiefel T.H., McCormack M., Fastnacht R.A., Ramesh R., Chen L.H. (1994). Thousandfold Change in Resistivity in Magnetoresistive La-Ca-Mn-O Films. Sci. New Ser..

[24] Ashok A., Kumar A., Ponraj J., Mansour S.A., Tarlochan F. (2021). Enhancing the electrocatalytic properties of LaMnO_3_ by tuning surface oxygen deficiency through salt assisted combustion synthesis. Catal. Today.

[25] Mefford J.T., Hardin W.G., Dai S., Johnston K.P., Stevenson K.J. (2014). Anion charge storage through oxygen intercalation in LaMnO_3_ perovskite pseudocapacitor electrodes. Nat. Mater..

[26] Martin-Rio S., Pomar A., Frontera C., Wang H., Manzorro R., Magén C., Balcells L., Mestres N., Martinez B. (2022). Spin to charge conversion in chemically deposited epitaxial La_0.9_MnO_3_ thin films capped with Pt. J. Mater. Chem. C.

[27] Manchón-Gordón A.F., Sánchez-Jiménez P.E., Blázquez J.S., Perejón A., Pérez-Maqueda L.A. (2023). Structural, Vibrational, and Magnetic Characterization of Orthoferrite LaFeO_3_ Ceramic Prepared by Reaction Flash Sintering. Materials.

[28] Cao E., Chu Z., Wang H., Hao W., Sun L., Zhang Y. (2018). Effect of film thickness on the electrical and ethanol sensing characteristics of LaFeO_3_ nanoparticle-based thick film sensors. Ceram. Int..

[29] Bidrawn F., Kim G., Aramrueang N., Vohs J.M., Gorte R.J. (2010). Dopants to enhance SOFC cathodes based on Sr-doped LaFeO_3_ and LaMnO_3_. J. Power Sources.

[30] Shi Z., Li H., Zhang L., Cao Y. (2022). Improved photocatalytic activity of LaFeO_3_ with doping Mn^3+^ ions and modifying Pd^2+^ ions for photoreduction of CO_2_ into CH_4_. J. Power Sources.

[31] Gu Z.F., Xu Y.J., Hong B., Xu J.C., Han Y.B., Jin H.X., Jin D.F., Peng X.L., Gong J., Ge H.L. (2023). Enhanced visible-light photocatalytic activity of g-C_3_N_4_/LaFeO_3_ heterojunctions for the removal of tetracycline hydrochloride. Diam. Relat. Mater..

[32] Lazarević Z.Ž., Jovalekić Č., Milutinović A., Romčević M., Romčević N.Ž. (2012). Preparation and Characterization of Nano Ferrites. Acta Phys. Pol. A.

[33] Ahmadpour G., Samardak A., Korochentsev V.V., Osmushko I.S., Samardak V., Komissarov A.A., Shtarev D.S., Samardak A.S., Ognev A.V., Nasirpouri F. (2021). Microstructure, composition and magnetic properties of Nd-(Fe_1-x_Co_x_)-B oxide magnetic particles synthesized by Pechini-type chemical method. Adv. Powder Technol..

[34] Lau L.N., Lim K.P., Ishak A.N., Awang Kechik M.M., Chen S.K., Ibrahim N.B., Miryala M., Murakami M., Shaari A.H. (2021). The Physical Properties of Submicron and Nano-Grained La_0.7_Sr_0.3_MnO_3_ and Nd_0.7_Sr_0.3_MnO_3_ Synthesised by Sol–Gel and Solid-State Reaction Methods. Coatings.

[35] Hernandez E., Sagredo V., Delgado G.E. (2015). Synthesis and magnetic characterization of LaMnO_3_ nanoparticles. Rev. Mex. Fis..

[36] Abazari R., Sanati S., Saghatforoush L.A. (2014). A unique and facile preparation of lanthanum ferrite nanoparticles in emulsion nanoreactors: Morphology, structure, and efficient photocatalysis. Mater. Sci. Semicond. Process..

[37] Shihua D., Tianxiu S., Xiaojing Y., Xiaobing L., Guobiao L. (2012). The dielectric properties of BaTiO_3_ based ceramics co-doped with Bi/Mn. Ceram. Int..

[38] Kasenov B.K., Kasenova S.B., Sagintaeva Z.I., Baisanov S., Lu N.Y., Nukhuly A., Kuanyshbekov E.E. (2023). Heat Capacity and Thermodynamic Functions of Titanium-Manganites of Lanthanum, Lithium and Sodium of LaLi_2_TiMnO_6_ and LaNa_2_TiMnO_6_. Molecules.

[39] Kasenov B.K., Kasenova S.B., Sagintaeva Z.I., Kuanyshbekov E.E., Mukhtar A.A., Kakenov K.S. (2022). Thermodynamics and Electrophysics of New LaCaCuZnMnO_6_ Copper–Zinc Manganite of Lanthanum and Calcium. High Temp..

[40] Dubey A., Sanjuán I., Andronescu C., Lupascu D.C. (2024). Bi site doped Ferroelectric BiFe_0.95_Mn_0.05_O_3_ Nanoparticles for Hydrogen Evolution Reaction. ChemCatChem.

