# Supplementary material for: Synthesis, Structure, and Electrophysical and Electrochemical Properties of Novel Composite La0.9MnO3-LaFeO3
Source: Molecules. 2024 Dec 31;30(1):132. doi: 10.3390/molecules30010132 (PMC11722482; doi:10.3390/molecules30010132)
Supplement: Supplementary file 1 [file molecules-30-00132-s001.zip › molecules-3349620-supplementary.pdf]

Supporting Information

Synthesis, structure, electrophysical and electrochemical properties of novel composite $La_{0.9}MnO_3$-$LaFeO_3$

Mukhametkali Mataev[1], Zamira Sarsenbaeva[1,*], Bahadir Keskin[2], Marzhan Nurbekova[1,*], Amangeldi Meldeshov[1], Zhanar Tursyn[1], Karima Seitbekova[1]

[1] Kazakh National Women's Teacher Training University, Gogol, 114/1, Almaty, Kazakhstan, 050000; mataev.m@qyzpu.edu.kz, (M.M.); meldeshov.a@qyzpu.edu.kz (A.M.); janar.tursyn@gmail.com, (Zh.T.); karimaseitbekova91@gmail.com, (K.S.).

[2] Yildiz Technical University, Faculty of Arts & Science, Istanbul, Turkey; TR34220; bahadirkeskin@gmail.com, (B.K.).

***** Correspondence: sarsenbayeva.zamira@qyzpu.edu.kz, (Z.S.); nurbekova.m@gmail.com, (M.N.).

Experimental sections

Materials: The following reagents were used: manganese (II) nitrate ($Mn(NO_3)_2{\cdot}xH_2O$, Buchs, Switzerland); iron (III) nitric acid crystalline hydrate with 9-water salt ($Fe(NO_3)_3{\cdot}9H_2O$, TU 6-09-02-553-96); lanthanum (III) nitrate with water 6-crystalline hydrate ($La(NO_3)_3{\cdot}6H_2O$, TU 6-09-4676-83); citric acid ($C_6H_8O_7$) (GOST 908-79) and ethylene glucol ($C_2H_6O_2$) (GOST 10164-75).

Preparation of $La_{0.9}MnO_3$-$LaFeO_3$ nanocomposite. During the synthesis of $La_{0.9}MnO_3$-$LaFeO_3$, varying precursor ratios were evaluated, including 9:1, 87:13, and 85:15. The 87:13 composition was selected for detailed investigation due to its favorable yield and reproducibility. For the synthesis, stoichiometric quantities of the precursors were measured as follows: 3.77 g of $La(NO_3)_3{\cdot}6H_2O$, 3.51 g of $Fe(NO_3)_3{\cdot}9H_2O$ and 1.58 g of $Mn(NO_3)_2{\cdot}xH_2O$. To prepare salt solutions, 10 mL of distilled water was added. To facilitate the synthesis, 2.0 g of citric acid (added in a 1:1.5 molar ratio to the total metal cations) and 2.72 mL of ethylene glycol (density: 1.1 g/mL) were included. Citric acid acted as a chelating agent, while ethylene glycol promoted polymerization, ensuring the formation of a homogeneous gel and contributing to the successful synthesis of the desired phase. The resulting gel was dried at 120°C for 12 hours and the porous product was milled and fired at 600-1200°C for 6 hours. As a result, a powder of perovskite-like biphasic nanocomposite was synthesized.

Preparation of a tablet for electrophysical measurement:

The research of electrophysical properties (dielectric constant and electrical resistivity) was carried out by measuring the electrical capacitance of the samples on a commercially available LCR-800 instrument (Taiwan) at an operating frequency of 1 kHz in continuous thermostat mode in dry air with each fixed temperature maintenance time. The research of

electrophysical properties (dielectric permittivity and electrical resistance) was carried out by measuring the electrical capacity of the samples on a commercially available device LCR-800 (Taiwan) at an operating frequency of 1kHz continuously in dry air in thermostatic mode with holding time at each fixed temperature. Flat-parallel specimens in the form of disks with a diameter of 10 mm and thickness of 2-6 mm with binder additive (~1,5 %) were prefabricated. Pressing was carried out under pressure of 20 kg/cm^2. The obtained disks were fired in the laboratory furnace at 400 ºC for 6 hours. Then thorough double-sided grinding was carried out.

Preparation of working electrode:

The prepared electrocatalysts were tested in a conventional triple electrode (reference electrode: Ag/AgCl, counter electrode: platinum plate, working electrode: glassy carbon) system setup in an acidic solution of 0.5 M sulfuric acid using a Gamry electrochemical workstation (Reference 600 Potentiostat) to evaluate their electrocatalytic activity for bifunctional electrocatalytic activity. A glassy carbon electrode (GCE: 0.0314 cm^{-2}) was used as the working electrode. To clean the working electrode, the GCE was polished with alumina powder (0.05 μm) and then sonicated in a mixture of EtOH and H_2O (1:3) for 5 min. To prepare homogenized ink, 5 mg of $La_{0.9}MnO_3$-$LaFeO_3$ and 2 mg of carbon black composite was dispersed in 1 mL of H_2O and further sonicated for 30 min. Then 15 μL of homogenized catalytic ink and 10 μL of Nafion-117 were dripped onto the cleaned GCE surface and allowed to dry at room temperature, respectively. Electrochemical impedance spectroscopy (EIS) and LSV were recorded for HER in 0.5 mol/L H_2SO_4 in the corresponding potential range.

Characterization:

Manganite and ferrite can be synthesized using different sol-gel methods; in this study, the

Pechini method was chosen to produce a double-phase composite. For the X-ray diffraction (XRD) analysis, we used a Rigaku MiniFlex 600 X-ray diffraction system from Rigaku Corporation in Tokyo, Japan. The system utilized CuKα radiation with a range of 2θ 3–120 °C, a step size of 2θ 0.01–0.02 °, and a time per step of 0.3–0.5 °C. A nickel monochromator was employed to capture the diffraction, and the data was analyzed using the PDXL2 databases. The average crystal size of $La_{0.9}MnO_3$-$LaFeO_3$ were calculated using Debye–Scherrer's formula. Fourier-transform infrared (FT-IR) spectroscopy was conducted using a Bruker ALPHA instrument (Ettlingen, Germany) under ambient conditions. The analysis was performed within the spectral range of 4000–400 cm^{-1}, employing KBr pellets as the medium. The resolution was set to 1 cm^{-1}, and to enhance the signal-to-noise ratio, 32 scans were averaged for each measurement. The finely powdered $La_{0.9}MnO_3$-$LaFeO_3$ was subjected to FESEM to examine its surface morphology and to determine its elemental composition. The analysis was performed using a Thermo Scientific Apreo 2 S LoVac (USA). The elemental composition was assessed through energy-dispersive X-ray spectroscopy (EDS), which was integrated with the SEM instrument. FESEM provided high-resolution surface measurements at 500 nm. The research of electrophysical properties (dielectric constant and electrical resistivity) was carried out by measuring the electrical capacitance of the samples on a commercially available LCR-800 instrument (Taiwan) at an operating frequency of 1 kHz in continuous thermostat mode in dry air with each fixed temperature maintenance time. Electrophysical measurements of $La_{0.9}MnO_3$-$LaFeO_3$ in the range 293-483 K and frequencies equal to 1, 5, and 10 kHz were carried out on the LCR-800 setup. The polarization method is a systematic and effective tool to investigate the electrochemical activity of electrocatalysts. These curves were recorded from 0.2-0.95 V vs. Ag/AgCl (reference electrode) at a scan

rate of 9.99 mV/s in 0.5 M H_2SO_4 using a three-probe electrode system. EIS is an effective method to determine the electrochemical characteristics of surface catalysts packing interfaces, etc. Electrocatalytic water splitting-one of the industries specializing in the production of high purity hydrogen where EIS is used to correlate trends that measure charge transfer activity (Rct). An electrochemically equivalent cell consists of the following elements: solution (Rs), charge transfer resistance (Rct) and Warburg(W) elements, double electrical layer capacitance (Cdl) and constant phase (CPE). EIS for $La_{0.9}MnO_3$-$LaFeO_3$ at different overpotentials in 0.5 M H_2SO_4, the data are fitted to the simplified equivalent circuit shown in the inset, and the fit results are plotted as solid traces.

Figures

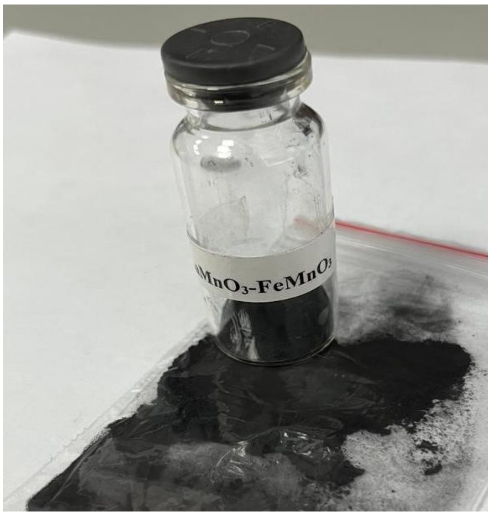

Figure S1. Photo images of the $La_{0.9}MnO_3$-$LaFeO_3$ nanocomposite powder

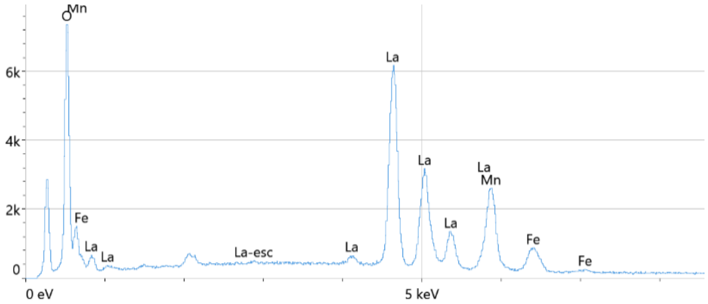

(a)

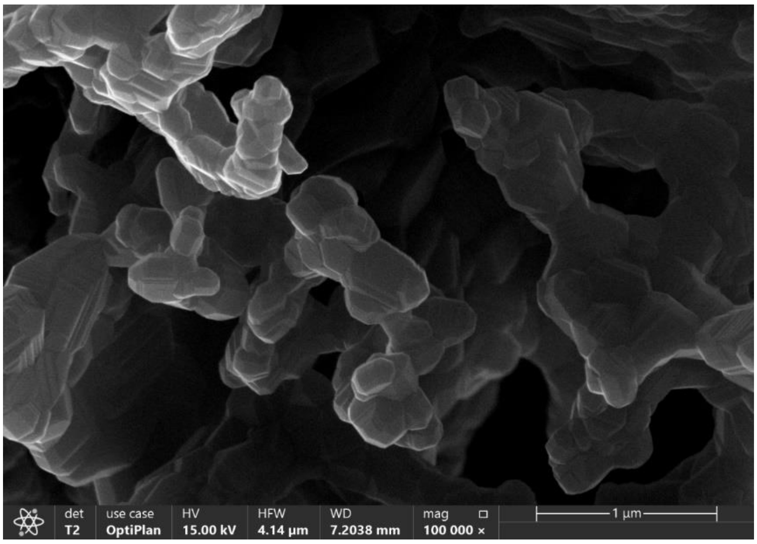

(b)

Figure S2. EDS point analysis results (a) and XRSEM images (b) of $La_{0.9}MnO_3$-$LaFeO_3$.

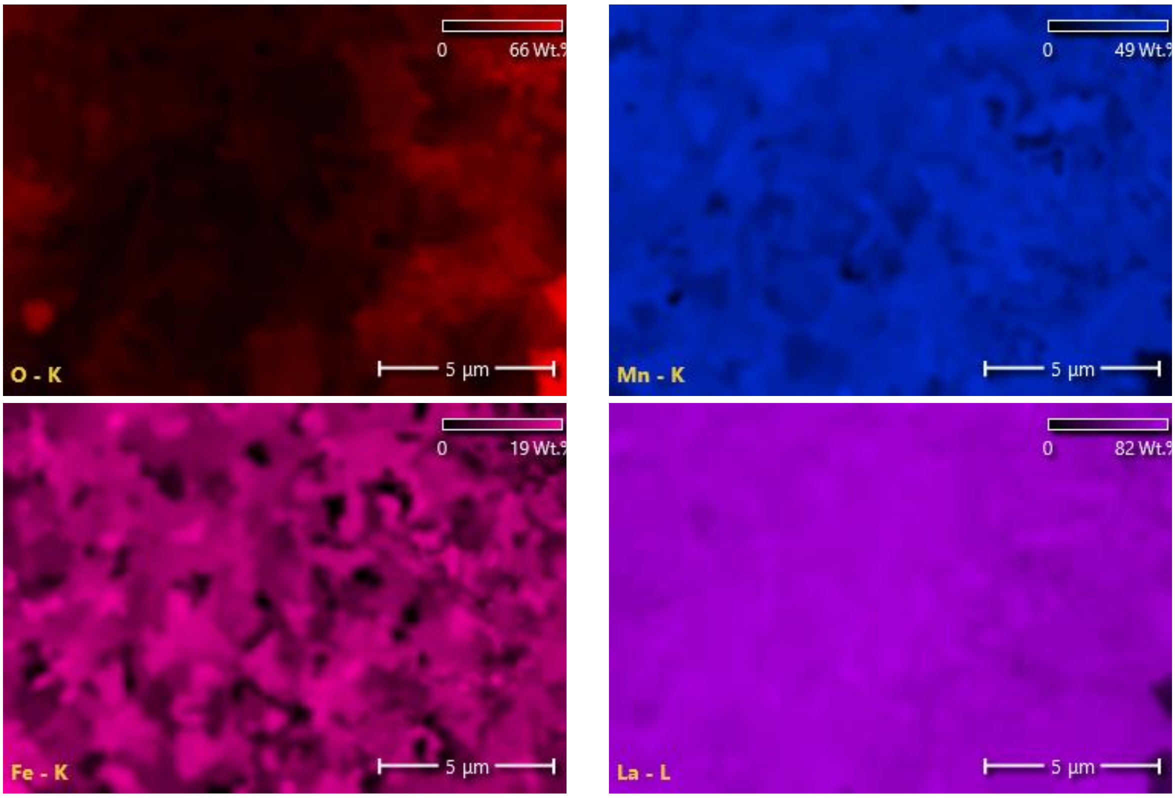

(a)

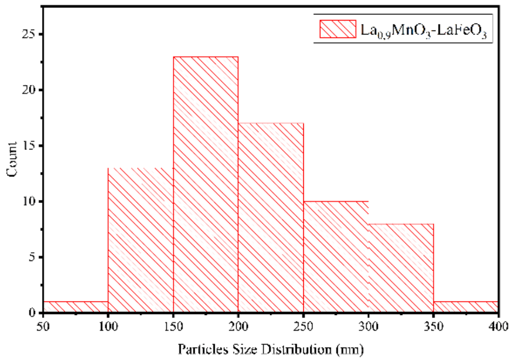

(b)

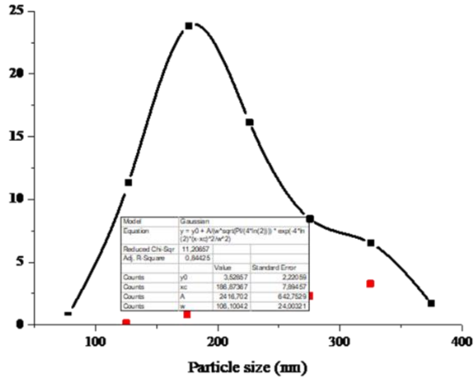

(c)

Figure S3 Mapping analysis results of $La_{0.9}MnO_3$-$LaFeO_3$ (a). Histogram of the particle size distribution of the samples determined from FESEM micrographs of manganite-ferrite composite (b,c).

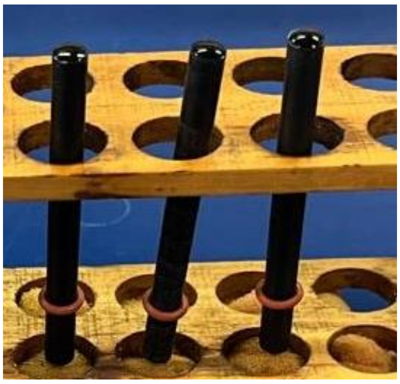

Figure S4. Photo images of the GCE modified with $La_{0.9}MnO_3$-$LaFeO_3$ composite material.
